# Perception–Adoption Gap of an AI Dietary Management App in Real-World Dining Settings: A Field Study

**DOI:** 10.3390/nu18162640

**Published:** 2026-08-12

**Authors:** Shupeng Mai, Jinji Xu, Zihan Hu, Chengdi Shan, Yuqi Zhao, Hongwei Liu, Qi Song, Zhenni Zhu

**Affiliations:** 1 Division of Health Risk Factors Monitoring and Control, National Health Commission Specialty Laboratory of Food Safety Risk Assessment and Standard Development, Shanghai Municipal Center for Disease Control and Prevention, Shanghai 200336, China; maishupeng@scdc.sh.cn (S.M.); songqi@scdc.sh.cn (Q.S.); 2 School of Public Health, Fudan University, Shanghai 200032, China; 22301020040@m.fudan.edu.cn (J.X.); zhhu24@m.fudan.edu.cn (Z.H.); 23301020107@m.fudan.edu.cn (Y.Z.); hwliu23@m.fudan.edu.cn (H.L.); 3 Department of Food and Nutrition Hygiene, Huangpu District Center for Disease Control and Prevention, Shanghai 200001, China; hpyyws@163.com

**Keywords:** artificial intelligence, dietary management, usability, user acceptance, real-world study

## Abstract

**Background/Objectives**: Although efficacious in randomized trials, the real-world adoption of AI-driven dietary management applications remains uncertain across diverse dining contexts and populations. **Methods**: This field-based observational study was conducted over 18 days at three real-world dining sites in Shanghai, China, enrolling 181 participants stratified into three groups based on food service style and customer attribute. A cross-sectional survey was administered on day 9, followed by a 9-day prospective usage tracking period. **Results**: After adjusting for sex, Group 2 (staff cafeteria with fixed-portion dishes) had the highest adjusted mean usability score at 71.20 (*p* < 0.001). Group 3 (community canteen) had the highest mean scores for information quality (16.57, *p* = 0.03) and perceptions of intended use in nutrition (12.01, *p* = 0.08). However, Group 3 recorded zero active usage sessions despite favorable initial perceptions. **Conclusions**: Favorable user perceptions of this AI-driven dietary management tool did not automatically translate into adoption. Scenario-specific usability and digital divide constraints define the boundary of real-world efficacy; moreover, AI may amplify existing dietary self-management behaviors rather than creating them de novo.

## 1. Introduction

The integration of artificial intelligence (AI) into healthcare is an evolving frontier [1]. Though most AI tools reported in medical literature target clinical applications, direct-to-consumer (DTC) mobile health apps have emerged as a rapidly growing domain [2], particularly in the fields of nutritional assessment and dietary management. Mounting evidence suggests that DTC apps have the potential to optimize daily food choices and intake estimation [3,4]. Additionally, the growing trend of leveraging AI to generate personalized dietary recommendations and analytics has expanded the integration of AI in the DTC field [5].

Despite this momentum, DTC apps face challenges in real-world implementation. The optimal business model for individually tailored DTC health management apps remains unclear [2]. This is largely due to uncertainties surrounding ideal scenarios and target populations—a challenge highlighted for DTC tools.

Nowadays, the study of healthcare integrated with AI has been transitioning from proof-of-concept in efficacy and effect toward later-stage evaluation of real-world adoption, user acceptance, trust, and efficiency. Our research team previously developed a mobile app powered by AI for an individual’s nutritional assessment and recommendation and studied its effects on the dietary improvements of individuals [6,7]. However, despite demonstrated effect, the real-world deployment of such tools remains uncertain, particularly given the heterogeneity of catering scenarios and user populations.

The current study aimed to conduct a later-stage, real-world transition of an AI-driven app for personalized dietary management. The objectives were to study the perception and acceptance of the app among target users and explore applicable scenarios on a real-world operational scale.

## 2. Materials and Methods

### 2.1. Settings and Study Design

This was a field-based real-world observational study conducted over 18 days to explore perception and acceptance following the use of an AI-driven personalized dietary management app. The study design comprised two integrated data streams: a cross-sectional perception survey and a short-term usage tracking period.

Two staff cafeterias and one community canteen in Shanghai, China, which served approximately 760 regular diners daily, were selected as the study fields.

The fieldwork was carried out in three sequential phases. Phase 1 was eight days before the field survey, when the app was introduced in the cafeteria or canteen. At the beginning of the eight days, the introduction and instructions for the app were provided both in four physical banner stands in highly visible locations of dining areas and through the messaging app. The app was officially available to all consumers. Phase 2 was the questionnaire survey day. Those who were regular diners and users of the app were encouraged to complete the questionnaire survey, with a gift incentive for their participation. Phase 3 was a nine-day follow-up to collect data on app use among the participants recruited in Phase 2. For usage tracking, a “usage session” was strictly defined as the completion of a comprehensive dietary record within the application.

### 2.2. Participants

The survey was conducted from August to December 2025 in three dining sites in Shanghai, China. The participants were enrolled from among those who regularly dined at the cafeterias or canteen and had previously used the app at least once.

This study was approved by the Ethics Committee of Shanghai Municipal Center for Disease Control and Prevention on 9 June 2025 (No. 2025-52). The study was conducted in accordance with the ethical principles of the Declaration of Helsinki. Written informed consent was obtained from all individual participants.

### 2.3. Group Classification

Participants were stratified into three groups, primarily based on food service style and customer attribute. The participants in Group 1 were working-age employees who were having lunch in a buffet-style cafeteria that regularly offered 16 dish options per meal. Consumers paid for the food by weight through an automated checkout system and instantly received an electronic bill including the absolute weight of each dish they took. The participants in Group 2 were employees who were having lunch in a staff cafeteria that offered 7 dish options, with each in a fixed portion per meal. One meal selection involved meat, vegetables, and staple food. The participants in Group 3 were mainly older adults who were having lunch in a community canteen that offered 26 dish options, with each in a fixed portion per meal. Consumers had no limits on food selection.

The app’s dietary recording interface was adapted to accommodate the three food service styles. Dietary recording required users to select the dish names and input both the consumed weight and the discarded weight. The participants in Group 1 recorded the dish names and the actual weight of the dishes they selected, with each diet recording taking approximately 40 s on average. The participants in Groups 2 and 3 recorded the dish names and the number of servings of the dishes they selected, completing each diet recording in less than 20 s.

### 2.4. Enrollment

A total of 203 consumers who were regular diners and had used the app to record at least one meal during Phase 1 were invited to participate in the investigation and completed an electronic questionnaire. Eligible participants were recruited directly from the canteen in which they regularly dined and were classified accordingly; therefore, they were not randomly allocated to the three groups. Of these, 22 were excluded due to low-quality responses, specifically defined as selecting the identical option for all items within the questionnaires. Finally, a total of 181 participants, with 57 in Group 1, 79 in Group 2, and 45 in Group 3, were enrolled in the current study.

### 2.5. Function of the AI-Driven App

**Figure 1:**
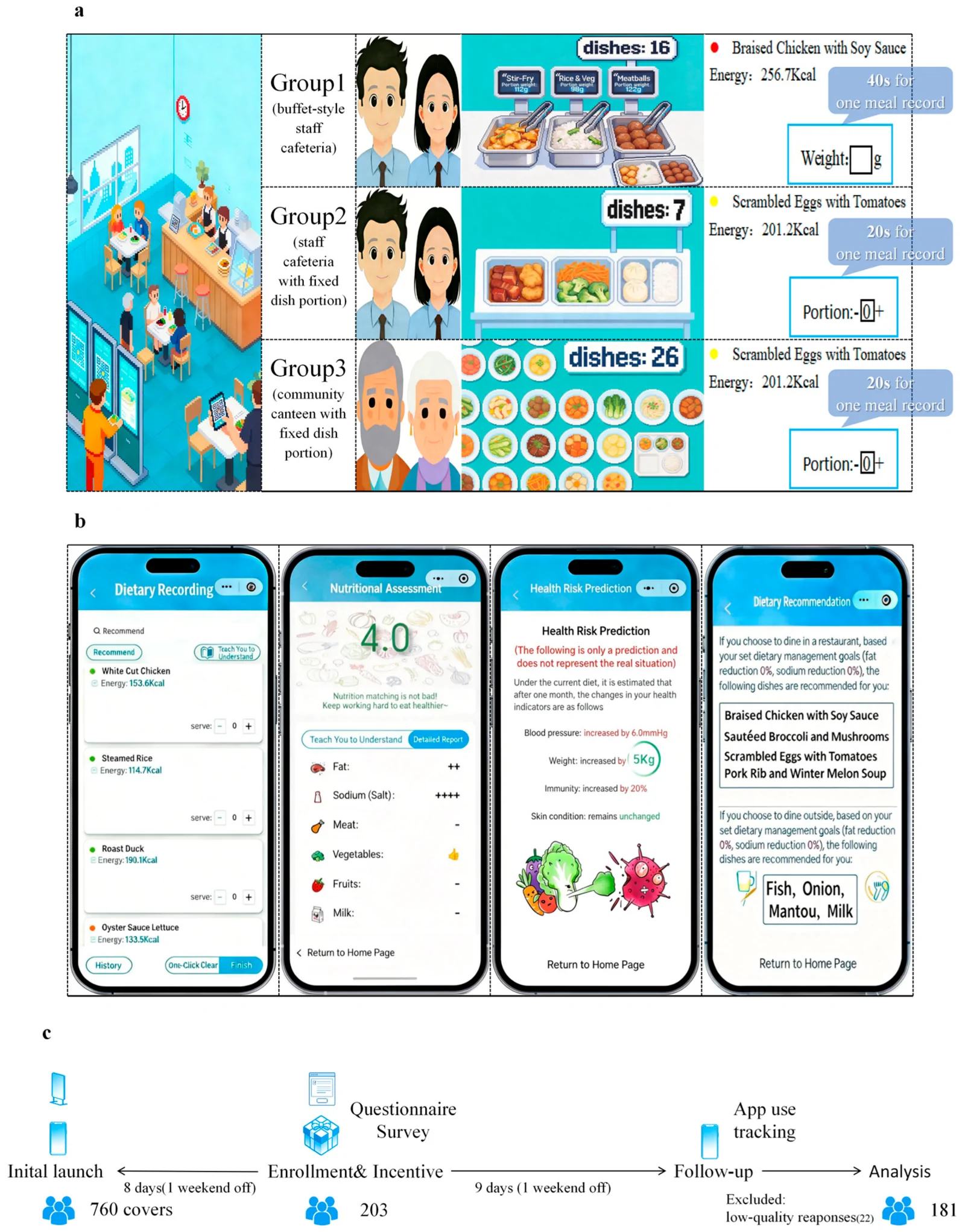
Study design and flowchart of the study. (**a**) Illustrates group classification by customer attribute, food service style, and dietary recording interface; (**b**) demonstrates the main interfaces of the app, including dietary recording, nutritional assessment, health risk prediction and dietary recommendations; (**c**) the study flow chart.

This AI-driven app was primarily designed for consumers dining in cafeterias that use standardized or semi-standardized recipes and cooking procedures. It had been proven effective for dietary self-management and optimizing individuals’ dietary structure in the previous study [7]. It was premised on the pre-collection of recipes and enabled users to quickly record the portion served and the leftovers for each dish. The AI algorithm included two parts. The first was a discriminative model that evaluated individual food and nutrient intake based on the user’s biological features. The other was a recommendation algorithm that, through time-series analysis of personal dietary records, generated personalized food suggestions integrating the user’s food preferences and health requirements [8] (Figure 1).

### 2.6. Preparation of Recipe Data

All three catering scenarios adopted a central kitchen catering model, with all dishes prepared in standardized cooking protocols. All dish information was collected under a standardized framework: the food types and weighing weights of raw materials and condiments were recorded first, followed by data calibration and integration into the recipe database. Integrated with the preloaded Chinese food composition database [9,10] in the data platform, it could then generate dish-specific nutritional labels and perform post-meal nutrition evaluations.

### 2.7. Dietary Recording and Nutritional Information

The participants were required to input self-biographic features (including age, sex, height, weight, physical activity level, etc.) before accessing the full functions of the app. The participants received real-time personalized nutrition information by recording the dishes they took and the proportion of leftovers of each dish.

The AI algorithm generated three kinds of personalized nutrition information: nutritional assessment, health risk prediction, and dietary recommendations. Nutritional assessment illustrated whether food and nutrient intakes were inadequate, adequate, or excessive according to the Chinese Dietary Guidelines. Health risk prediction provided an estimation of changes in health indicators after one month, assuming the continuation of current dietary patterns. This estimation was based on a general dietary response model, further refined by a calibration model for continuous individual-level data. An algorithm processed continuous individual dietary intake and health data alongside the Chinese Dietary Guidelines to generate personalized dietary recommendations. These recommendations then integrated personal dietary preferences and nutritional requirements to guide the selection of dishes or foods for the next meal.

For data governance, the app utilized the mobile phone number as the unique identifier for each user. Additionally, blockchain technology was integrated into the system architecture to ensure strict data confidentiality and security throughout the process. Additional details of the AI architecture, structured input features, and preliminary interface testing are provided in Supplementary File S6.

### 2.8. Perception and Acceptance Assessment

Questionnaire Survey: All data were collected via self-reported electronic questionnaires at the study cafeterias or canteen. The System Usability Scale (SUS) consisted of ten questions, which was applied to assess the perceived usability of the app. Higher scores indicated superior usability [11]. In the current sample, the SUS demonstrated good internal consistency (Cronbach’s α = 0.87). An Information Quality Questionnaire (IQQ) comprised seven questions, in which four questions were adapted from the Information Quality section in the end-user version of the Mobile Application Rating Scale questionnaire [12], and the other three were designed to measure users’ perceptions of the app’s intended use in the nutrition field. The adapted IQQ also showed excellent internal consistency (Cronbach’s α = 0.93). The questionnaires had been validated for healthcare application use and employed a 5-point Likert scale ranging from strongly disagree (1) to strongly agree (5) as its response options [13,14], and both of them were translated and culturally adapted following standard forward–backward translation procedures; the complete SUS and IQQ instruments are provided in Files S1 and S2.

Post-Use Survey: Acceptance of the app was regarded as the use frequency of the app following an incentive-based questionnaire survey. Dietary records in the app and interface interaction logs of each participant during the survey days were obtained from the system platform.

### 2.9. Statistical Analysis

All participants’ characteristics were categorical variables; thus, they were summarized as frequencies with corresponding percentages. The Kruskal–Wallis H test was used to analyze age distribution, whereas Fisher’s exact test was employed to examine differences in categorical variables (i.e., sex and occupation).

Analysis of covariance (ANCOVA) was performed to quantify the independent effect of dining scenarios on outcome variables, with sex designated as a covariate. SUS and IQQ scores were reported as mean values with 95% confidence intervals (CIs). Additionally, graphical illustrations were generated to depict the click count of each app functional interface and the actual post-incentive app usage frequency across the three dining scenarios.

All statistical analyses were conducted using R (Version 4.4.2, R Project for Statistical Computing). The FSA package was applied for KW tests, and the emmeans package was used for the ANCOVA model. Statistical significance was defined as a two-tailed *p*-value < 0.05.

## 3. Results

### 3.1. Participant Characteristics

A total of 181 participants were included in the final analysis. The number (percentage) of participants in the age ranges of 31–40 and 41–50 was 60 (33.1%) and 49 (27.1%), respectively; 50.3% were female. Statistically significant differences in age, sex, and occupational distribution were observed across the three groups (Table 1).

### 3.2. Usability Evaluation of the App

After adjusting for sex, Group 2 had the highest adjusted mean of the SUS total score at 71.20 (*p* < 0.001) among the three groups (Table 2). Post hoc tests indicated statistically significant differences between Group 2 and 1.

### 3.3. Perceptions of the App

After adjusting for sex, Group 3 had the highest mean scores at 16.57 for information quality and 12.01 for perceptions of intended use in the nutrition field (*p* = 0.03 and *p* = 0.08, respectively; Table 3). Statistically significant differences were also found between Groups 3 and 1 in the visual information part (*p* = 0.02) and credibility of source part (*p* = 0.02). In the perceptions of intended use in the nutrition field section, statistically significant differences were observed among groups in the meal planning part.

### 3.4. Acceptance of the App

**Figure 2:**
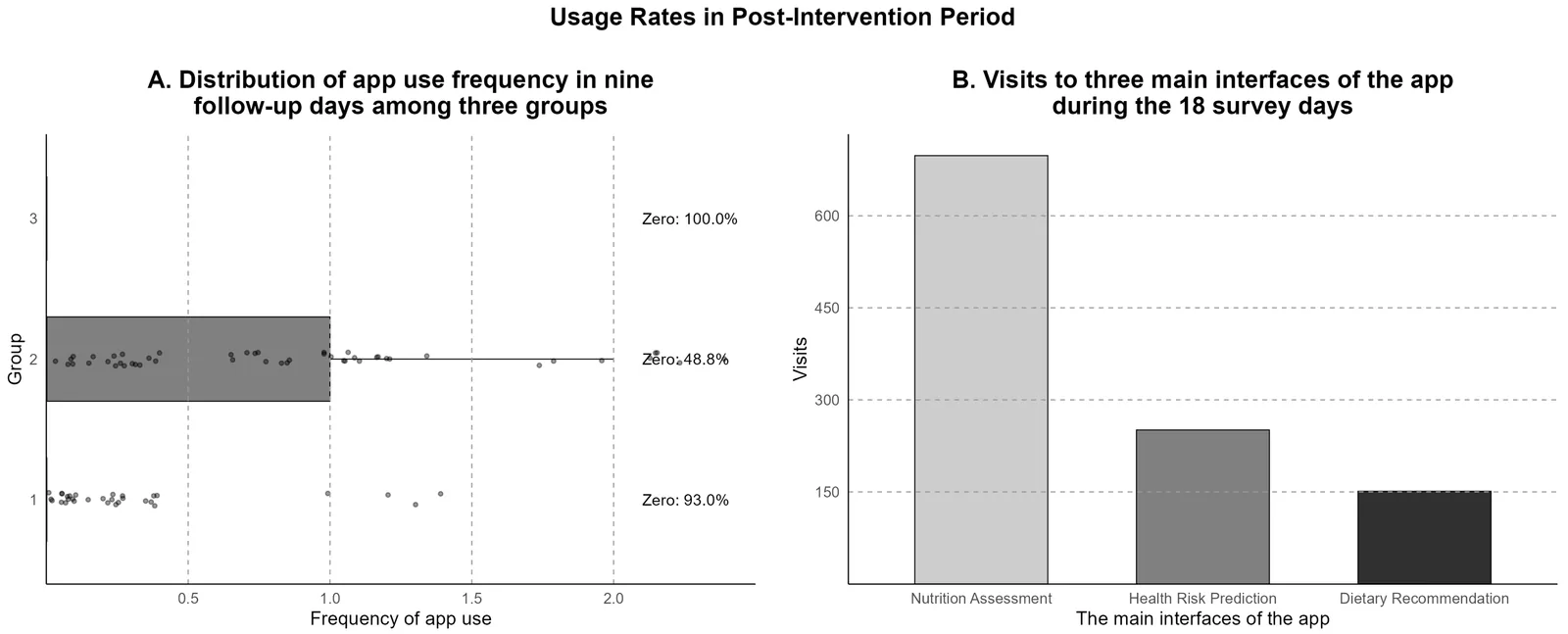
The app use during the survey period among the participants. (**A**) depicts the app use frequency during the 9-day follow-up period (excluding the enrollment and incentive day). Mean frequency of app use per participant was defined as the ratio of total use frequency to the number of participants within each group. (**B**) shows the visits to three main interfaces of the app among all participants in Groups 2 and 3 during the whole 18 survey days.

During the 9-day follow-up period, the distribution of app usage frequency varied notably across the three groups (Figure 2A). Group 2 demonstrated the highest overall engagement, with a median frequency of one session (IQR: 0–1). In stark contrast, no meal records were obtained for any participant in Group 3 during this follow-up window, resulting in a 100% zero-session rate. Group 1 also exhibited a remarkably high proportion of non-users, with 93.0% of participants recording zero sessions. Regarding feature-specific engagement over the entire 18-day study period, the nutritional assessment interface remained the most frequently visited module in both Group 2 and Group 3 (Figure 2B). Detailed interface visit data was unavailable for Group 1. Group-specific app-use retention patterns are presented in File S6.

## 4. Discussion

This study revealed a consistent perception–adoption gap across all three dining scenarios: favorable initial attitudes toward the AI dietary app did not translate into sustained real-world use. While scenario-specific usability constraints, the digital divide, and absent self-managed dietary behavior operated as distinct mechanisms, all three converged on the same outcome—disengagement.

We found that even an extra few seconds in the diet recording process can negatively impact perceived usability. Despite being matched for age, the participants in Group 2 assigned significantly higher ratings for the app’s perceived usability, whereas those in Group 1 reported considerably lower satisfaction levels. Within China’s healthcare framework, dietitians are not prevalent [15]. The population had little exposure to nutritional consultation, and as a result, dietary reporting was an unfamiliar practice to most. The main reason for the current result was probably that the time taken for dietary recording was different between the two groups. Those in the cafeteria scenario (Group 1) had to take 40 s or so to record the exact weight of each dish before receiving AI-generated nutrition information, which was, on average, 20 s longer than those in the fixed-portion serving scenario (Group 2), who only needed to record the number of dishes consumed. Integration of AI technologies primarily aims to facilitate a burdensome workload [16,17]. In contrast to data collection via smartwatch or camera [18,19], dietary recording often requires user cooperation [4]. Some participants likely mistook the weight input column for number of servings, which led to incorrect entries. This error might have undermined the perceived usability of the feature for this user group. Future designs of comparable apps should take efforts to minimize dietary recording time, for example, preferably integrating with checkout systems to reduce manual entry.

The participants generally recognized that AI was integrated into personalized dietary management, especially the older ones, who demonstrated the most positive perception. It was beyond our expectations that the participants of Group 3 perceived this AI-generated information as more useful than other groups did, despite previously mentioning they had some operational barriers within the app. This aligned with previous studies that the digital divide lessened to some extent among older adults during the COVID-19 pandemic, potentially increasing their openness to digital health tools [20]. The perceived usefulness among the older participants might be linked to their greater need for health management, as they were more vulnerable to chronic diseases. However, the digital divide likely impeded their access to this AI-powered nutrition management tool in our study. In other words, it hindered their utilization of advanced health management services. An obvious gap persisted: a high demand for health management coexisted with a lack of sufficient skills to effectively cooperate with digital tools. As the older participant represented a key demographic for this DTC app, the future design could prioritize elder-friendly features such as elder-friendly onboarding and larger UI elements [21].

Synthesizing the scenario-specific findings above, we turn to an integrated analysis. Between the perception and acceptance of AI, there might lie the development of self-managed dietary behavior. Our findings indicated that the staff cafeteria, serving fixed-portion dishes, proved to be an ideal scenario for implementing the app. The other two scenarios, however, presented more challenges. A clear dichotomy emerged as a post-use among the participants: none of the groups became frequent users, working-age individuals in Group 2 transitioned to moderate use, the counterparts in Group 1 transitioned to occasional use, and the participants in Group 3 discontinued use. Though DTC agentic AI has garnered significant interest and demonstrates clear long-term potential in the healthcare field, there is still a substantial innovation–implementation gap to be bridged [22]. We found an obvious factor at play beyond the adaptation of AI technology in the current study. DTC apps were usually designed for self-managed health. However, when it comes to shifting from “seeing a doctor” to “self-managed health”, our participants had yet to develop the corresponding behavioral patterns, unlike the deeply ingrained behavior of “seeing a doctor”. This might explain why forging a viable business model in the DTC space, compared to the established clinical paradigm, requires further exploration and refinement [23].

This study has several limitations that should be considered. First, the relatively small sample size might result in insufficient statistical power, which could affect the stability and generalizability of the findings. Second, the study grouping was based on a combination of customer attributes and restaurant serving type. It was possible to introduce bias when interpreting the differences between the groups. Although education level and health status were measured, they were not included as additional covariates because the primary analysis was intended to preserve the naturally occurring sociodemographic profiles of the dining settings; nevertheless, residual confounding cannot be fully excluded, and the influence of these factors warrants further investigation in future studies using larger samples and complementary analyses with covariate adjustment. Furthermore, data on page visits were only available for Groups 2 and 3. The absence of this data for Group 1 might lead to an incomplete analysis of overall behavioral patterns, and some conclusions could therefore be partial or less accurate.

## 5. Conclusions

Favorable perception does not guarantee adoption of an AI dietary management tool as an effective nutrition intervention. Scenario-specific usability and digital divide constraints define the boundary of real-world efficacy. AI may amplify existing dietary self-management behaviors rather than creating them de novo. For AI to advance from technical proof-of-concept to sustainable nutrition intervention, its success metric needs to shift from algorithmic accuracy to self-management behavioral sustainability.

## Figures and Tables

**Table 1:** Characteristics of the participants.

		Participants, No(%)			
		Group 1	Group 2	Group 3	*p* Value ^a^
		(*n* = 57)	(*n* = 79)	(*n* = 45)	
Age					
	18–30	15 (26.3)	23 (29.1)	0	<0.001
	31–40	23 (40.4)	27 (34.2)	10 (22.2)	
	41–50	13 (22.8)	25 (31.6)	11 (24.4)	
	51–60	5 (8.8)	4 (5.1)	12 (26.7)	
	60–	1 (1.8)	0	12 (26.7)	
Sex					
	Female	13 (22.8)	51 (64.6)	27 (60.0)	<0.001
	Male	44 (77.2)	28 (35.4)	18 (40.0)	
Occupation					
	Managers	3 (5.3)	21 (26.6)	4 (8.9)	<0.001
	Professionals	39 (68.4)	52 (65.8)	1 (2.2)	
	Clerical support workers	4 (7.0)	2 (2.5)	12 (26.7)	
	Sales and Service workers	7 (12.3)	4 (5.1)	5 (11.1)	
	Plant and machine operators, and assemblers	1 (1.8)	0	0	
	Retired	3 (5.3)	0	23 (51.1)	

^a^ Kruskal–Wallis test was applied to identify the difference in age between groups, and Fisher’s exact test for sex and occupation.

**Table 2:** Usability evaluation of the app by groups ^a^.

		Group 1	Group 2	Group 3	*p* Value ^b^
Total score		60.91 (57.09 to 64.72) ^d^	71.20 (68.06 to 74.34) ^c^	65.48 (61.38 to 69.58) ^c,d^	<0.001
	I think that I would like to use this app frequently.	3.63 (3.41 to 3.85)	3.77 (3.59 to 3.95)	3.90 (3.66 to 4.13)	0.27
	I found the app unnecessarily complex.	3.01 (2.77 to 3.26) ^c^	2.36 (2.16 to 2.56) ^d^	2.82 (2.56 to 3.08) ^c^	<0.001
	I thought the app was easy to use.	3.59 (3.37 to 3.81)	3.94 (3.76 to 4.12)	3.82 (3.59 to 4.06)	0.06
	I think that I would need the support of a technical person to be able to use this app.	2.01 (1.82 to 2.20) ^c^	1.64 (1.49 to 1.80) ^d^	2.01 (1.81 to 2.21) ^c^	0.003
	The various functions in this app are well integrated.	3.77 (3.57 to 3.97)	3.93 (3.77 to 4.10)	4.04 (3.82 to 4.25)	0.21
	There were too many inconsistencies in this app.	3.06 (2.83 to 3.30) ^c^	2.48 (2.28 to 2.67) ^d^	2.63 (2.38 to 2.89) ^c,d^	0.002
	I would imagine that most people would learn to use this system very quickly.	3.70 (3.49 to 3.90) ^d^	4.05 (3.88 to 4.22) ^c^	3.90 (3.68 to 4.12) ^c,d^	0.04
	I found the app very cumbersome to use.	2.89 (2.63 to 3.14) ^c^	2.15 (1.94 to 2.36) ^d^	2.76 (2.49 to 3.04) ^c^	<0.001
	I felt very confident using the app.	3.47 (3.22 to 3.73)	3.70 (3.49 to 3.91)	3.82 (3.55 to 4.09)	0.19
	I needed to learn a lot of things before I could get going with this app.	2.82 (2.57 to 3.06) ^c^	2.28 (2.08 to 2.49) ^d^	3.05 (2.78 to 3.31) ^c^	<0.001

Note: ^a^ Each question used a 5-point Likert scale ranging from strongly disagree (1) to strongly agree (5) as its response options; ^b^ ANCOVA was conducted, followed by Bonferroni post hoc tests for pairwise comparisons after adjustment for sex; ^c,d^ post hoc analysis was conducted. Superscripts with the same letter indicate no statistically significant difference between two groups, whereas different letters denote a significant difference. A group labeled with both superscripts b and c indicates no statistically significant difference from the other two groups.

**Table 3:** Perceptions of the app by groups ^a^.

		Group 1	Group 2	Group 3	*p* Value ^b^
Information Quality		15.24 (14.58 to 15.90) ^c^	15.81 (15.26 to 16.35) ^c,d^	16.57 (15.87 to 17.28) ^d^	0.03
	Quality of information: The app content is correct, well written, and relevant to the goal of the app.	3.79 (3.60 to 3.98)	3.89 (3.74 to 4.05)	4.10 (3.90 to 4.30)	0.08
	Quantity of information: The information within the app is comprehensive and concise.	3.80 (3.61 to 3.99)	3.97 (3.81 to 4.13)	4.08 (3.87 to 4.29)	0.15
	Visual information: Visual explanation of concepts through images is clear, logical, correct.	3.77 (3.59 to 3.96) ^c^	3.94 (3.79 to 4.09) ^c,d^	4.17 (3.97 to 4.36) ^d^	0.02
	Credibility of source: The information within the app seems to come from a credible source.	3.87 (3.71 to 4.04) ^c^	4.00 (3.87 to 4.14) ^c,d^	4.22 (4.05 to 4.40) ^d^	0.02
Perceptions of intended use in nutrition field		11.06 (10.46 to 11.66)	11.24 (10.75 to 11.74)	12.01 (11.37 to 12.66)	0.08
	For meal planning: The app is helpful for planning a nutrition-balanced lunch.	3.63 (3.41 to 3.85)	3.67 (3.49 to 3.85)	4.00 (3.76 to 4.23)	0.05
	For accessing nutrition information: The app increases my awareness of daily energy and nutrient intake.	3.71 (3.50 to 3.92)	3.91 (3.74 to 4.08)	4.07 (3.84 to 4.29)	0.08
	For weight management: The app provides support for managing my weight.	3.72 (3.48 to 3.96)	3.66 (3.46 to 3.86)	3.95 (3.70 to 4.21)	0.19

Note: ^a^ Each question used a 5-point Likert scale ranging from strongly disagree (1) to strongly agree (5) as its response options; ^b^ ANCOVA was conducted, followed by Bonferroni post hoc tests for pairwise comparisons after adjustment for sex; ^c,d^ post hoc analysis was conducted. Superscripts with the same letter indicate no statistically significant difference between two groups, whereas different letters denote a significant difference. A group labeled with both superscripts b and c indicates no statistically significant difference from the other two groups. Item-level sensitivity analyses using ordinal logistic regression are reported in File S3: Tables S1 and S2. Effect-size estimates and Bonferroni-adjusted pairwise comparisons are reported in File S4: Tables S3 and S4. Sensitivity analyses including all 203 respondents are provided in File S7: Tables S5 and S6.

## Data Availability

The datasets used and analyzed during the current study are available from the corresponding author upon reasonable request. The datasets are not publicly available due to participant privacy considerations.

## Supplementary Materials

The following supporting information can be downloaded at: https://www.mdpi.com/article/10.3390/nu18162640/s1, File S1: System Usability Scale (SUS) Questionnaire; File S2: Information Quality Questionnaire (IQQ); File S3: Ordinal Logistic Regression: Table S1: Sensitivity analysis of individual SUS items using ordinal logistic regression; Table S2: Sensitivity analysis of individual information quality and perceived intended-use items using ordinal logistic regression; File S4: Effect size post hoc comparisons: Table S3: Partial eta squared and Bonferroni-adjusted post hoc pairwise comparisons for SUS outcomes; Table S4: Partial eta squared and Bonferroni-adjusted post hoc pairwise comparisons for information quality and perceived intended-use outcomes; File S5: App use retention; Figure S1: Kaplan–Meier curves of app-use retention across the three groups; File S6: Methodological detail; Supplementary method S1: Detailed methodological information on participant selection, AI tool architecture, input features, and preliminary validation; File S7: Full sample sensitivity analysis; Table S5: Usability evaluation of the app by groups including all 203 respondents; Table S6: Perceptions of the app by groups including all 203 respondents.

## Abbreviations

The following abbreviations are used in this manuscript:

AI	Artificial Intelligence
ANCOVA	Analysis of Covariance
CI	Confidence Interval
DTC	Direct-to-Consumer
IQQ	Information Quality Questionnaire
SUS	System Usability Scale
uMARS	User Version of the Mobile Application Rating Scale
SE	Standard error
