# Supplementary material for: Perception–Adoption Gap of an AI Dietary Management App in Real-World Dining Settings: A Field Study
Source: Nutrients. 2026 Aug 12;18(16):2640. doi: 10.3390/nu18162640 (PMC13516353; doi:10.3390/nu18162640)
Supplement: Supplementary file 1 [file nutrients-18-02640-s001.zip › Supplement 1.pdf]

The System Usability Scale (SUS)

Please tick the box corresponding to your attitude towards the following statement.

	Strongly disagree 1	2	3	4	Strongly agree 5
1. I think that I would like to use this app frequently.					
2. I found the app unnecessarily complex.					
3. I thought the app was easy to use.					
4. I think that I would need the support of a technical person to be able to use this app.					
5. The various functions in this app are well integrated.					
6. There were too many inconsistencies in this app.					
7. I would image that most people would learn to use this system very quickly.					
8. I found the app very cumbersome to use.					
9. I felt very confident using the app.					
10. I needed to learn a lot of things before I could get going with this app.					
