# Supplementary material for: Perception–Adoption Gap of an AI Dietary Management App in Real-World Dining Settings: A Field Study
Source: Nutrients. 2026 Aug 12;18(16):2640. doi: 10.3390/nu18162640 (PMC13516353; doi:10.3390/nu18162640)
Supplement: Supplementary file 1 [file nutrients-18-02640-s001.zip › Supplement 2.pdf]

The Information Quality Questionnaire(IQQ)

Please tick the box corresponding to your attitude towards the following statement.

	Strongly disagree				Strongly agree
**Information Quality**					
1. Quality of information: The app content is correct, well written, and relevant to the goal of the app.	1	2	3	4	5
2. Quantity of information: The information within the app is comprehensive and concise.	1	2	3	4	5
3. Visual information: Visual explanation of concepts through images is clear, logical, correct.	1	2	3	4	5
4. Credibility of source: The information within the app seems to come from a credible Source.	1	2	3	4	5
**Perceptions of intended use in nutrition field**					
5. For meal planning: The app is helpful for planning a nutrition-balanced lunch.	1	2	3	4	5
6. For accessing nutrition information: The app increases my awareness of daily energy and nutrient intake.	1	2	3	4	5
7. For weight management: The app provides support for managing my weight.	1	2	3	4	5
