# Supplementary material for: Perception–Adoption Gap of an AI Dietary Management App in Real-World Dining Settings: A Field Study
Source: Nutrients. 2026 Aug 12;18(16):2640. doi: 10.3390/nu18162640 (PMC13516353; doi:10.3390/nu18162640)
Supplement: Supplementary file 1 [file nutrients-18-02640-s001.zip › Supplyment 3.pdf]

Table S1. Sensitivity analysis of the SUS individual items using Ordinal Logistic Regression

Note: [a] overall *p* value; [b] *p* values for pairwise comparisons adjusted with the Bonferroni method.

	***p* value**[a]	**Contrast**	**OR**	***p* value pairwise**[b]
SUS				
I think that I would like to use this app frequently.	0.16	Group1-Group2	0.65(0.33 to 1.30)	0.44
		Group1-Group3	0.47(0.22 to 1.03)	0.14
		Group2-Group3	0.73(0.36 to 1.48)	0.65
I found the app unnecessarily complex.	<0.001	Group1-Group2	3.86(1.94 to 7.67)	<0.001
		Group1-Group3	1.50(0.69 to 3.29)	0.57
		Group2-Group3	0.39(0.19 to 0.81)	0.03
I thought the app was easy to use.	0.06	Group1-Group2	0.43(0.21 to 0.87)	0.05
		Group1-Group3	0.57(0.26 to 1.24)	0.33
		Group2-Group3	1.31(0.65 to 2.66)	0.73
I think that I would need the support of a technical person to be able to use this app.	0.006	Group1-Group2	2.86(1.39 to 5.88)	0.01
		Group1-Group3	1.13(0.50 to 2.58)	0.95
		Group2-Group3	0.40(0.19 to 0.84)	0.04
The various functions in this app are well integrated.	0.25	Group1-Group2	0.72(0.36 to 1.46)	0.64
		Group1-Group3	0.50(0.22 to 1.13)	0.22
		Group2-Group3	0.69(0.33 to 1.44)	0.59
There were too many inconsistencies in this app.	<0.001	Group1-Group2	3.81(1.91 to 7.58)	<0.001
		Group1-Group3	3.00(1.37 to 6.58)	0.02
		Group2-Group3	0.79(0.39 to 1.61)	0.79
I would image that most people would learn to use this system very quickly.	0.03	Group1-Group2	0.39(0.19 to 0.78)	0.02
		Group1-Group3	0.54(0.24 to 1.23)	0.31
		Group2-Group3	1.41(0.68 to 2.92)	0.62
I found the app very cumbersome to use.	<0.001	Group1-Group2	4.82(2.39 to 9.73)	<0.001
		Group1-Group3	1.57(0.72 to 3.41)	0.49
		Group2-Group3	0.33(0.16 to 0.69)	0.009
I felt very confident using the app.	0.21	Group1-Group2	0.68(0.35 to 1.32)	0.49
		Group1-Group3	0.51(0.24 to 1.09)	0.19
		Group2-Group3	0.75(0.38 to 1.49)	0.69
I needed to learn a lot of things before I could get going with this app.	<0.001	Group1-Group2	3.26(1.63 to 6.52)	0.002
		Group1-Group3	0.75(0.35 to 1.60)	0.74
		Group2-Group3	0.23(0.11 to 0.47)	<0.001

Table S2. Sensitivity analysis of Information Quality and Perceptions of Intended Use in the nutrition field individual items using Ordinal Logistic Regression

	p value[a]	Contrast	OR	*p* value pairwise[b]
Information Quality				
Quality of information: The app content is correct, well written, and relevant to the goal of the app.	0.05	Group1-Group2	0.67(0.33 to 1.39)	0.54
		Group1-Group3	0.35(0.15 to 0.82)	0.04
		Group2-Group3	0.52(0.25 to 1.11)	0.21
Quantity of information: The information within the app is comprehensive and concise.	0.07	Group1-Group2	0.53(0.26 to 1.11)	0.21
		Group1-Group3	0.39(0.17 to 0.90)	0.07
		Group2-Group3	0.72(0.34 to 1.53)	0.67
Visual information: Visual explanation of concepts through images is clear, logical, correct.	0.01	Group1-Group2	0.55(0.26 to 1.14)	0.25
		Group1-Group3	0.29(0.13 to 0.66)	0.01
		Group2-Group3	0.52(0.25 to 1.10)	0.20
Credibility of source: The information within the app seems to come from a credible Source.	0.02	Group1-Group2	0.65(0.31 to 1.36)	0.48
		Group1-Group3	0.30(0.13 to 0.71)	0.02
		Group2-Group3	0.47(0.22 to 1.00)	0.12
Perceptions of intended use in nutrition field				
For meal planning: The app is helpful for planning a nutrition-balanced lunch.	0.04	Group1-Group2	0.82(0.41 to 1.65)	0.85
		Group1-Group3	0.38(0.17 to 0.84)	0.05
		Group2-Group3	0.46(0.22 to 0.94)	0.08
For accessing nutrition information: The app increases my awareness of daily energy and nutrient intake.	0.05	Group1-Group2	0.57(0.28 to 1.17)	0.28
		Group1-Group3	0.35(0.15 to 0.81)	0.04
		Group2-Group3	0.61(0.29 to 1.29)	0.40
For weight management: The app provides support for managing my weight.	0.13	Group1-Group2	1.04(0.52 to 2.09)	0.99
		Group1-Group3	0.51(0.23 to 1.14)	0.23
		Group2-Group3	0.49(0.24 to 1.02)	0.13

Note:[a]overall *p* value;[b]*p* values for pairwise comparisons adjusted with the Bonferroni method.
