# Supplementary material for: Perception–Adoption Gap of an AI Dietary Management App in Real-World Dining Settings: A Field Study
Source: Nutrients. 2026 Aug 12;18(16):2640. doi: 10.3390/nu18162640 (PMC13516353; doi:10.3390/nu18162640)
Supplement: Supplementary file 1 [file nutrients-18-02640-s001.zip › Supplyment 4.pdf]

Table S3. Partial eta squared and adjusted post hoc pairwise comparisons for the SUS

	Partial Eta squared	contrast	Mean Difference	SE	p value[a]
Total score	0.085	Group1-Group2	-10.29	2.58	<0.001
		Group1-Group3	-4.58	2.88	0.34
		Group2-Group3	5.72	2.59	0.09
I think that I would like to use this app frequently.	0.015	Group1-Group2	-0.14	0.15	1.00
		Group1-Group3	-0.27	0.17	0.32
		Group2-Group3	-0.13	0.15	1.00
I found the app unnecessarily complex.	0.093	Group1-Group2	0.66	0.16	<0.001
		Group1-Group3	0.19	0.18	0.90
		Group2-Group3	-0.47	0.16	0.02
I thought the app was easy to use.	0.031	Group1-Group2	-0.35	0.15	0.06
		Group1-Group3	-0.23	0.16	0.48
		Group2-Group3	0.12	0.15	1.00
I think that I would need the support of a technical person to be able to use this app.	0.064	Group1-Group2	0.37	0.13	0.01
		Group1-Group3	0.00	0.14	1.00
		Group2-Group3	-0.37	0.13	0.01
The various functions in this app are well integrated.	0.018	Group1-Group2	-0.16	0.14	0.69
		Group1-Group3	-0.27	0.15	0.24
		Group2-Group3	-0.10	0.14	1.00
There were too many inconsistencies in this app.	0.071	Group1-Group2	0.58	0.16	0.001
		Group1-Group3	0.43	0.18	0.05
		Group2-Group3	-0.16	0.16	1.00
I would image that most people would learn to use this system very quickly.	0.036	Group1-Group2	-0.35	0.14	0.04
		Group1-Group3	-0.20	0.16	0.59
		Group2-Group3	0.15	0.14	0.82
I found the app very cumbersome to use.	0.113	Group1-Group2	0.74	0.17	<0.001
		Group1-Group3	0.12	0.19	1.00
		Group2-Group3	-0.61	0.17	0.002
I felt very confident using the app.	0.019	Group1-Group2	-0.23	0.17	0.57
		Group1-Group3	-0.35	0.19	0.22
		Group2-Group3	-0.12	0.17	1.00
I needed to learn a lot of things before I could get going with this app.	0.117	Group1-Group2	0.53	0.17	0.005
		Group1-Group3	-0.23	0.19	0.65
		Group2-Group3	-0.76	0.17	<0.001

Note:[a]*p* values for pairwise comparisons adjusted with the Bonferroni method.

Table S4. Partial eta squared and adjusted post hoc pairwise comparisons for the Information Quality and Perceptions of Intended Use

	Partial Eta squared	contrast	Mean Difference	SE	p value[a]
Information Quality	0.039	Group1-Group2	-0.57	0.45	0.61
		Group1-Group3	-1.34	0.50	0.02
		Group2-Group3	-0.77	0.45	0.26
Quality of information: The app content is correct, well written, and relevant to the goal of the app.	0.028	Group1-Group2	-0.10	0.13	1.00
		Group1-Group3	-0.31	0.14	0.09
		Group2-Group3	-0.21	0.13	0.30
Quantity of information: The information within the app is comprehensive and concise.	0.021	Group1-Group2	-0.17	0.13	0.56
		Group1-Group3	-0.28	0.15	0.16
		Group2-Group3	-0.11	0.13	1.00
Visual information: Visual explanation of concepts through images is clear, logical, correct.	0.045	Group1-Group2	-0.17	0.12	0.52
		Group1-Group3	-0.39	0.14	0.01
		Group2-Group3	-0.23	0.12	0.21
Credibility of source: The information within the app seems to come from a credible Source.	0.043	Group1-Group2	-0.13	0.11	0.77
		Group1-Group3	-0.35	0.13	0.02
		Group2-Group3	-0.22	0.11	0.15
Perceptions of intended use in nutrition field	0.029	Group1-Group2	-0.18	0.40	1.00
		Group1-Group3	-0.96	0.45	0.11
		Group2-Group3	-0.77	0.41	0.18
For meal planning: The app is helpful for planning a nutrition-balanced lunch.	0.033	Group1-Group2	-0.04	0.15	1.00
		Group1-Group3	-0.36	0.17	0.09
		Group2-Group3	-0.32	0.15	0.09
For accessing nutrition information: The app increases my awareness of daily energy and nutrient intake.	0.028	Group1-Group2	-0.20	0.14	0.46
		Group1-Group3	-0.36	0.16	0.07
		Group2-Group3	-0.16	0.14	0.83
For weight management: The app provides support for managing my weight.	0.019	Group1-Group2	0.06	0.16	1.00
		Group1-Group3	-0.23	0.18	0.60
		Group2-Group3	-0.29	0.16	0.22

Note:[a] *p* values for pairwise comparisons adjusted with the Bonferroni method.
