# Supplementary material for: Perception–Adoption Gap of an AI Dietary Management App in Real-World Dining Settings: A Field Study
Source: Nutrients. 2026 Aug 12;18(16):2640. doi: 10.3390/nu18162640 (PMC13516353; doi:10.3390/nu18162640)
Supplement: Supplementary file 1 [file nutrients-18-02640-s001.zip › Supplyment 6.pdf]

Detailed Methodological Information on Participant Selection, AI Tool Architecture, Input Features, and Preliminary Validation

1. Detailed Selection and Exclusion Criteria

Inclusion Criteria: individuals who had used the AI app to record at least one meal during the 8-day promotional period (Phase 1) and successfully completed the electronic questionnaire survey on Day 9 were enrolled.

Exclusion Criteria: Participants were excluded if they submitted low-quality questionnaire responses. Low quality was strictly defined as "straight-lining" behavior, specifically selecting the identical response option for all items within either the System Usability Scale (SUS) or the adapted Information Quality Questionnaire (IQQ).

2. AI Model Configuration and Architecture

The AI-driven dietary management tool was deployed as a WeChat Mini Program to ensure high accessibility. The core AI architecture consists of two primary modules:

Discriminative Model (Nutritional Assessment & Risk Prediction): The prior model was constructed using a baseline dataset comprising anthropometric and dietary records from 24 individuals over a 14-day period. To achieve personalized precision nutrition, the system employs a Bayesian model to perform posterior updates. It continuously integrates users' real-time uploaded dietary records and anthropometric data to generate individualized nutritional assessments and predict future health risk indicators.

Recommendation Model: The dietary recommendation algorithm utilizes Particle Swarm Optimization (PSO). Based on the outputs from the discriminative model and the user's historical dietary patterns, the PSO algorithm optimizes and generates tailored, quantitative dietary guidance for the user's subsequent meals.

3. "Prompt" Design and Input Features

Unlike generative text-based Large Language Models (LLMs) that rely on natural language prompts, the AI modules in this tool are driven by structured data inputs. In the context of our algorithmic framework, the "prompts" function as structured input parameters that trigger the Bayesian updates and PSO optimization. These input features include:

Dietary Record Specific types of consumed foods, exact consumed weights and discarded weights .

Anthropometric Inputs: Baseline user characteristics, including age, sex, height, weight, and physical activity level.

These structured variables serve as the mathematical inputs that dynamically drive the generation of personalized nutritional metrics and dietary recommendations.

4. Preliminary Testing and Validation Steps

Prior Efficacy Validation: Before conducting this 18-day real-world field study, the fundamental efficacy of this AI tool was validated in our previous research [https://doi.org/10.3390/nu16040565]. That study confirmed that the tool effectively assists the experimental group in weight management over observation period. Building upon this proven efficacy, the primary objective of the current study was to investigate

its deployment, usability, and adoption in real-world dining settings.

Pilot Testing for Interface Adaptation: Prior to the main 18-day field study, preliminary pilot testing was conducted to refine the dietary recording interface for the three distinct food service styles. This included optimizing the user interface to ensure that recording exact weights (Group 1) and fixed-portion servings (Groups 2 and 3) could be completed efficiently (averaging 40 seconds and <20 seconds, respectively), and verifying the stability of backend data synchronization across different catering scenarios.
