# Supplementary material for: Perception–Adoption Gap of an AI Dietary Management App in Real-World Dining Settings: A Field Study
Source: Nutrients. 2026 Aug 12;18(16):2640. doi: 10.3390/nu18162640 (PMC13516353; doi:10.3390/nu18162640)
Supplement: Supplementary file 1 [file nutrients-18-02640-s001.zip › Supplyment 7.pdf]

Table S5. Usability Evaluation of the App by Groups Including All 203 Respondents.

	Group 1	Group 2	Group 3	*p* value
Total score	61.58 (58.01 to 65.14)[a]	70.47 (67.57 to 73.37)[b]	64.78 (61.09 to 68.46)[a]	<0.001
I think that I would like to use this app frequently.	3.67 (3.46 to 3.89)	3.74 (3.57 to 3.91)	3.83 (3.61 to 4.05)	0.61
I found the app unnecessarily complex.	3.03 (2.80 to 3.26)[a]	2.36 (2.18 to 2.55)[b]	2.87 (2.63 to 3.11)[a]	<0.001
I thought the app was easy to use.	3.60 (3.39 to 3.81)	3.92 (3.76 to 4.09)	3.79 (3.57 to 4.00)	0.07
I think that I would need the support of a technical person to be able to use this app.	1.97 (1.79 to 2.14)[a],[b]	1.68 (1.54 to 1.83)[a]	2.02 (1.83 to 2.20)[b]	0.008
The various functions in this app are well integrated.	3.81 (3.62 to 4.00)	3.88 (3.73 to 4.04)	4.04 (3.85 to 4.24)	0.23
There were too many inconsistencies in this app.	3.06 (2.83 to 3.28)[a]	2.45 (2.26 to 2.63)[b]	2.70 (2.47 to 2.94)[a],[b]	<0.001
I would image that most people would learn to use this system very quickly.	3.74 (3.55 to 3.94)	4.03 (3.88 to 4.19)	3.89 (3.69 to 4.09)	0.08
I found the app very cumbersome to use.	2.84 (2.60 to 3.08)[a]	2.20 (2.00 to 2.40)[b]	2.71 (2.46 to 2.96)[a]	<0.001
I felt very confident using the app.	3.51 (3.26 to 3.75)	3.65 (3.45 to 3.84)	3.75 (3.49 to 4.00)	0.42
I needed to learn a lot of things before I could get going with this app.	2.80 (2.57 to 3.04)[a]	2.35 (2.16 to 2.54)[b]	3.09 (2.84 to 3.33)[a]	<0.001

Notes: [a,b]Post hoc analysis was conducted. Superscripts with the same letter indicated no statistically significant difference between two groups, whereas different letters denoted a significant difference. A group labeled with both superscripts b and c indicated no statistically significant difference from the other two groups.

Table S6. Perceptions of the App by Groups Including All 203 Respondents.

	Group 1	Group 2	Group 3	*p* value[b]
Information Quality	15.32 (14.69 to 15.95)[a]	15.83 (15.31 to 16.34)[a],[b]	16.53 (15.88 to 17.18)[b]	0.04
Quality of information: The app content is correct, well written, and relevant to the goal of the app.	3.82 (3.65 to 4.00)	3.89 (3.75 to 4.04)	4.08 (3.90 to 4.26)	0.12
Quantity of information: The information within the app is comprehensive and concise.	3.82 (3.64 to 4.01)	3.98 (3.83 to 4.13)	4.09 (3.90 to 4.28)	0.15
Visual information: Visual explanation of concepts through images is clear, logical, correct.	3.80 (3.62 to 3.97)[a]	3.96 (3.81 to 4.10)[a],[b]	4.16 (3.98 to 4.34)[b]	0.02
Credibility of source: The information within the app seems to come from a credible Source.	3.88 (3.72 to 4.04)[a]	4.00 (3.86 to 4.13)[a],[b]	4.20 (4.04 to 4.37)[b]	0.02
Perceptions of intended use in nutrition field	11.18 (10.63 to 11.74)	11.23 (10.78 to 11.68)	12.03 (11.46 to 12.61)	0.05
For meal planning: The app is helpful for planning a nutrition-balanced lunch.	3.67 (3.46 to 3.87)	3.68 (3.52 to 3.85)	4.01 (3.80 to 4.22)	0.03
For accessing nutrition information: The app increases my awareness of daily energy and nutrient intake.	3.76 (3.57 to 3.96)	3.88 (3.71 to 4.04)	4.07 (3.86 to 4.27)	0.11
For weight management: The app provides support for managing my weight.	3.75 (3.53 to 3.97)	3.67 (3.49 to 3.85)	3.96 (3.73 to 4.19)	0.15

Notes: [a,b]Post hoc analysis was conducted. Superscripts with the same letter indicated no statistically significant difference between two groups, whereas different letters denoted a significant difference. A group labeled with both superscripts b and c indicated no statistically significant difference from the other two groups.
